# An outbreak of cholera in Medipally village, Andhra Pradesh, India, 2013

**DOI:** 10.1186/s41043-015-0021-1

**Published:** 2015-07-24

**Authors:** Chengappa K. Uthappa, Ramesh R. Allam, Chava Nalini, Deepak Gunti, Prasada R. Udaragudi, Geetha P. Tadi, Manoj V. Murhekar

**Affiliations:** 1 SHARE India, Hyderabad, Andhra Pradesh India; 2 Directorate of Health, Ministry of Health and Family Welfare, Hyderabad, Andhra Pradesh India; 3 National Institute of Epidemiology, R-127, Tamil Nadu Housing Board, Ayapakkam, Chennai, Tamil Nadu 600 077 India

## Abstract

**Background:**

Cholera continues to remain endemic in over 50 countries and has caused large epidemics with around 3–5 million cases occurring every year in Asia alone. In India, cholera is endemic in many states. However, etiological information and age-specific incidence related to cholera outbreaks is limited. In November 2013, district authorities reported a cluster of diarrheal disease among residents of Medipally to the state surveillance unit. We investigated this cluster to confirm its etiology, describe its magnitude, identify potential risk factors, and make recommendations for control.

**Findings:**

A house-to-house active search was conducted to identify cases of acute diarrhea and collect information on drinking water source. Drinking water samples were collected from common water sources and sampled households to test for bacteriological quality. Ten stool samples were collected for culture. A matched case–control study was conducted to identify the risk factors. A total of 138 case-patients of diarrhea (Attack rate: 11.5/100; Population: 15 1,200) and 1 death (Case Fatality Ratio: 0.72/100) were identified. Five of the 10 stool samples were culture positive for V. cholerae, serogroup O1 El Tor. Drinking water from the overhead tank [Adjusted OR (AOR): 31.94, 95 % CI: 7.3-139.5] was associated with risk of developing illness.

**Conclusions:**

This outbreak affected nearly 11% of the village population and was due to contamination of the main drinking water source. Outbreaks such as this can be prevented by constructing the drain away from the water pipelines and by monitoring regular chlorination of drinking water source and inspection of pipelines for damage.

## Introduction

Diarrheal diseases constitute an important cause of morbidity and mortality globally, especially in the developing economies [1]. Cholera is an acute secretory diarrhea caused by the ingestion of Gram-negative bacterium *Vibrio cholerae* present in contaminated water or food [2]. Cholera leads to severe dehydration and death if left untreated [3]. The absence or shortage of safe drinking water, and lack of proper sanitation, facilitates the transmission of cholera [4]. Cholera continues to remain endemic in over 50 countries and has caused large epidemics [5]. Although cholera is vastly under-reported, WHO has estimated that around 3–5 million cases occur every year, predominantly in Asia and Africa [6]. In India, cholera is endemic in many states. During 1997–2006, India reported 68 outbreaks with 37,783 cases and 84 deaths [7]. More than 90 % of the cholera cases reported during these outbreaks were from the state of Orissa, West Bengal, Andaman and Nicobar Islands, Assam, and Chhattisgarh [7]. Limited capacities in disease surveillance, reluctance of authorities to report cholera cases for fear of societal repercussion that there has been a breakdown in sanitation and in the supply of safe water and delay in reporting in outbreaks have contributed largely to the underreporting of cases and hindered prompt response to cholera outbreaks in India [7].

Andhra Pradesh is a rapidly urbanizing state in southern India with a population of over 84 million people; sporadic outbreaks of acute diarrheal disease are a common phenomenon in this region. However, etiological information and age-specific incidence related to such outbreak in this region is limited. On November 3, 2013 the Medical and Health Officer of Mahabubnagar district in Andhra Pradesh informed the state surveillance unit about a cluster of Acute Diarrheal Disease (ADD) with one death at Medipally village (n = 1200). A team from the State Integrated Disease Surveillance Unit initiated epidemiological investigation on 5 November 2013 to i) confirm the etiology ii) describe the characteristics of the outbreak by time, place and person iii) identify risk factors for the outbreak, and iv) propose recommendations to contain the spread.

## Materials and methods

### Descriptive epidemiology

**Fig. 1:**
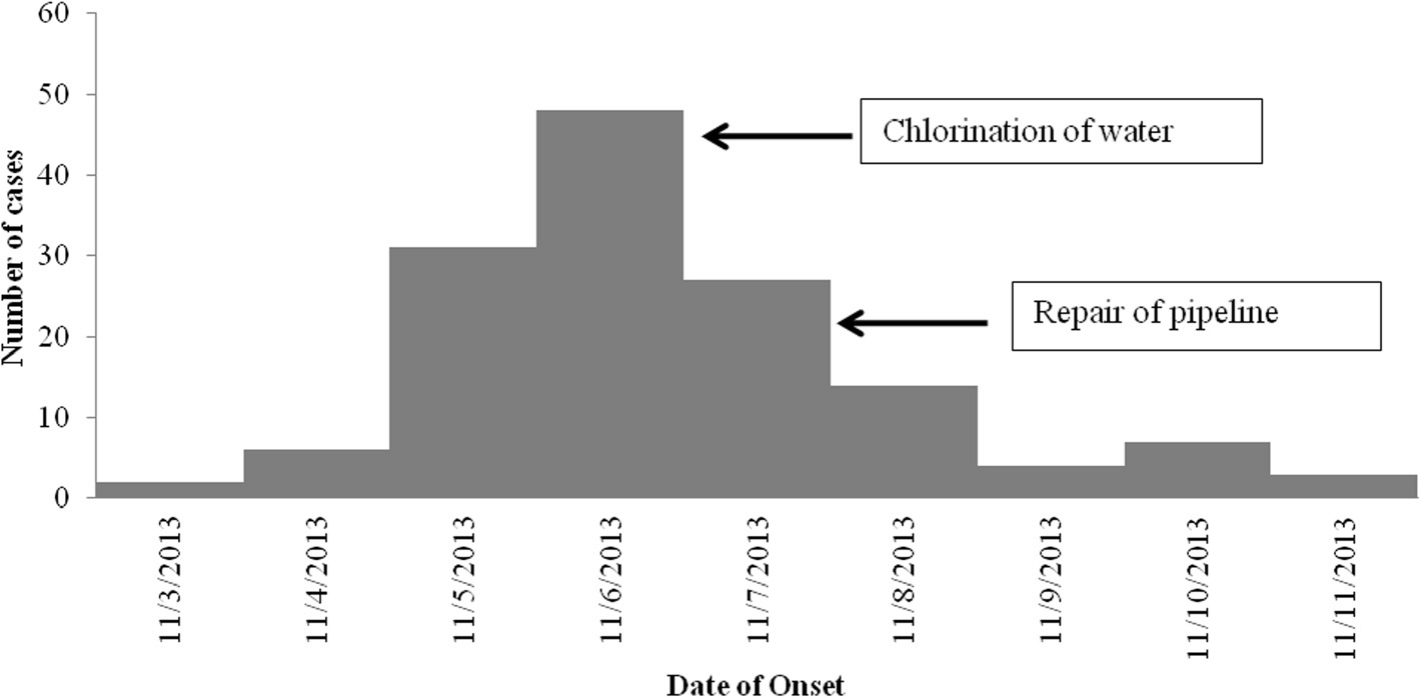
Cases of diarrhea by date of onset, Medipally, Mahabubnagar, Andhra Pradesh, India, November 2013

We reviewed the district Integrated Disease Surveillance Project (IDSP) weekly data for the years 2010, 2011 and 2012 to confirm the existence of outbreak. We defined a case of acute diarrhea as the occurrence of > =3 loose stools in a day among the residents of Medipally village since November 3, 2013. Six trained health workers conducted a house-to-house active search in the village to identify suspected cases and collected information about age, gender, place of residence and date of onset of the illness from them. We also collected information about the source of drinking water in the village. We analyzed the data to describe the characteristics of cases over time by using an epidemic curve (Fig. 1). We prepared a spot map to understand the geographical distribution of case-patients and calculated the attack rates by age and sex.

### Environmental investigation

We inspected all public drinking water sources, water pipelines and collected information about sanitation practices in the village. Drinking water samples were collected from the common water sources and few households with cases of acute diarrhea and tested for bacteriological quality. Houses were sampled to represent all localities of the village and different water sources.

### Laboratory investigation

Rectal swabs were collected from 10 case-patients during the initial two days of investigation and were transported using alkaline peptone water to the state referral laboratory at the Institute of Preventive Medicine, Hyderabad for microbiological investigations. The samples were incubated for six hours in alkaline peptone water and cultured on thio-sulphate-citrate-bile salt-sucrose (TCBS) agar followed by microscopy, biochemical analysis, and serotyping. Drinking-water samples collected from the village were tested for coliform count by membrane-filtration method at the district water quality-monitoring laboratory.

### Analytical epidemiology

Review of the descriptive epidemiological findings led us to suspect that the illness was associated with drinking water supplied from overhead tank. To test this hypothesis, we conducted a matched case control study. All case-patients meeting the case definition of acute diarrhea occurred during the outbreak were considered as cases. We selected one age-matched (±5 years) healthy subject from the same neighborhood as that of the case-patient as control.

Using a pre-tested, closed-ended questionnaire, we collected information on socio-demographic characteristics, source/s of drinking water and personal hygienic practices from cases and controls. The investigation was exempted from the ethical committee clearance as it was part of the state level emergency public health response to the outbreak. Informed consent was obtained from all the cases-patients and controls before collecting the above information.

The data was analyzed using Epi Info version 3.5.1 (Centers for Disease Control and Prevention, Atlanta, USA). We calculated the matched odds ratios (mOR) and their 95 % confidence intervals (CIs) associated with the independent variables. All the independent variables that were significantly associated (*p* < 0.05) were included into the conditional logistic model to calculate adjusted odds ratios (AOR).

## Findings

### Descriptive epidemiology

On review of the weekly surveillance data of Mahabubnagar district for the years 2010–2012, we confirmed an increase in incidence of diarrheal cases and ruled out recent population influx or any change in the reporting system. On house-to-house search, we identified 138 case-patients of diarrhea with an attack rate of 11.5 %. One case-patient died (case fatality ratio: 0.72 %). All age groups were affected, with the higher attack rates among children aged < =5 years. Both genders were equally affected (Table 1). The first case was reported on 3^rd^ November 2013, and subsequently number of cases peaked on 6^th^ November and then declined by 11^th^ November (Fig. 1) following chlorination of the overhead tank and repair of the damaged pipeline. The shape of the epidemic curve suggested a common-source, continuous exposure. The spot-map of the village indicated clustering of cases in area-1 of the village. (Data not shown).

**Table 1:** Age-group-specific attack rate of diarrhea cases in Medipally, Mahabubnagar, Andhra Pradesh, India, November-2013

Population groups		No. of cases	Population	Attack rate (%)
Age group (Years)	0-5	19	42	45
	06-14	18	54	33
	15-29	25	109	23
	30-44	31	222	14
	45-59	24	460	5
	Above 60	21	313	7
Gender	Male	73	624	12
	Female	65	576	11
	Total	138	1200	11.5

### Laboratory investigation

Five of the ten rectal swabs were culture positive for *V. cholerae*, O1 El Tor. Five of seven water samples (one from the overhead tank, one from the water-tap receiving water from the overhead tank, and three from stored water from households) had the Most Probable Number (MPN) count higher than the permissible level (10 MPN/100 ml) [8] and hence were considered unsafe for drinking. Two samples (one from the functional ground water hand pump and one form the stored water from household) had no coliforms and hence were potable.

### Environmental investigation

The village received water from two main sources: First, ground water that was pumped into the overhead tank and supplied to the households through public taps. The second source was from ground water hand pump. The village was divided in to two areas based on drinking water supply: area-1 exclusively received water from the overhead tank, and area-2 received water exclusively from the ground water hand pump. The attack rate in area-1 (10.6 %; 94 of 887) was more than that of area-2 (7.2 %; 44 of 613, *p* = 0.02). An open drain, measuring about 150 m was running alongside the water pipelines pumping ground water into the overhead tank. On close inspection of the drinking water pipelines, we observed that the main pipeline had a breakage near the valve and there was stagnation of sewage water around the pipeline. The sanitary conditions in the village were poor; most population practiced open defecation, the drainage system was of open type and was clogged within the vicinity of dwelling.

### Analytical epidemiology

We recruited 138 matched case–control pairs in the case–control study. Case patients and controls were not different with respect to their socio-economic characteristics. The odds of disease were 29 times higher among those who drank water from overhead tank as compared to those drinking water from ground hand pumps (mOR: 29, 95 % CI: 8.45-176.7, Table 2). The practice of drinking boiled water (mOR: 0.16, 95 % CI: 0.073-0.339) and regularly washing hands before eating (mOR: 0.23, 95 % CI: 0.12-0.44) were associated with lower odds of developing diarrhea (Table 2).

**Table 2:** Distribution of case–control sets (n = 138) according to exposure status, Cholera outbreak, Medipally, Andhra Pradesh, 2013

Exposure	Number of case–control pairs				Matched odds ratio	95 % Confidence interval	
	Concordant for exposure status		Discordant for exposure status				
	All exposed	All unexposed	Case exposed	Case unexposed			P
Gender	62	62	11	3	3.67	1.08- 6.36	0.06
Education-Illiterate	53	63	8	14	0.57	0.22- 1.35	0.14
Monthly family income < Rs 3000	31	71	19	17	1.12	0.57-2.17	0.87
Employment status unemployed	122	2	6	8	0.75	0.24-2.21	0.42
Drinking exclusive Overhead tank water	56	22	58	2	29.00	8.45- 176.71	0.00
Presence of house flies	69	38	22	9	2.44	1.14- 5.58	0.03
< Mean household members (4)	59	16	22	41	0.54	0.31- 0.89	0.01
Attended fair	0	129	7	2	3.50	0.77- 24.58	0.18
Practices							
Regularly drink boiled water	6	76	8	48	0.17	0.07- 0.33	0.01
Wash hands before eating	46	34	11	47	0.23	0.11- 0.44	0.01

On conditional logistic regression, drinking water from overhead tank (AOR: 31.94, 95 % CI: 7.3-139.5) was significantly associated with occurrence of diarrhea while drinking boiled water and regularly washing hands before eating were protective (Table 3).

**Table 3:** Factors associated for transmission of Cholera in conditional logistic regression

Exposure factors	Adjusted Odds Ratio (AOR)	95 % CI	P
Drink boiled water	0.03	0.01-0.13	0.001
Presence of house flies	2.15	0.56-8.15	0.264
Drinking exclusive Overhead tank water	31.94	7.31-139.53	0.001
< Mean house hold members (4)	0.86	0.38-1.96	0.72
Washed hands before eating	0.13	0.04-0.41	0.001

## Discussion

A large outbreak of cholera occurred in Medipally village, Mahabubnagar district affecting 11 % of the village population. Several factors supported our finding that the outbreak was due to contamination of main water source: First, the epi-curve suggested a common source epidemic; the sudden increase in the number of cases could be because of a recent breach in the pipeline. There was stagnation of sewage around the pipelines. This could have led to suction of the sewage into the water pipeline [9]. Second, the environmental survey suggested that area-1 which received drinking water from the overhead tank had higher attack rate compared to the other area. Third, on bacteriological examination of different water sources of the village, fecal contamination was present in sources that were supplied from the overhead tank. Fourth, drinking water from overhead tank was associated with higher odds of disease.

In India, cholera outbreaks in areas supplied with piped water systems that suffer from breaks in quality system and maintenance, including lack of chlorination are frequently reported [5, 9, 10]. The Millennium Development Goals consider piped water as an improved water source. Though Medipally village had piped water supply, water was not regularly chlorinated and non-maintenance of the pipeline increased the risk of acquiring infection. People from Area-2 were also consuming water from overhead tank intermittent though that was not the predominant source.

Our investigation suffered from one main limitation: we did not attempt *Vibrio cholerae* isolation from drinking water, which could have conclusively proved our hypothesis that the outbreak was due to contamination of main water source.

Based on the findings of our investigation, village authorities repaired the damaged water pipelines and chlorinated the overhead tank. To prevent such outbreaks in future, we recommended constructing the drain away from the water pipelines and regular chlorination of the overhead tank. The village panchayat, using the village sanitation funds has initiated the work for a new drain.
